# Research status and hotspots of medication safety in older adults: A bibliometric analysis

**DOI:** 10.3389/fpubh.2022.967227

**Published:** 2023-01-05

**Authors:** Chuantao Xie, Yanhong Gu, Yanan Wang, Feixia Ni, Yumei Li

**Affiliations:** ^1^Shanghai Fifth People's Hospital, Fudan University, Shanghai, China; ^2^School of Nursing, Fudan University, Shanghai, China; ^3^Center of Community-Based Health Research, Fudan University, Shanghai, China

**Keywords:** patient safety, medication safety, aged, polypharmacy, inappropriate prescribing, bibliometric analysis, research trends, hotspots

## Abstract

**Background:**

Medication safety is a significant concern in healthcare. Research on medication safety for older adults has taken a broad approach, resulting in a range of proposals. At this juncture, it is necessary to identify the main contributors and establish the current developmental status of the principal research topics.

**Objective:**

This study sets out to summarize the state-of-the-art in medication safety for older adults, identifying significant achievements, key topics, and emerging trends.

**Methods:**

The Web of Science Core Collection (WOSCC) database was searched for relevant documentation over the interval 1st January 2000 to 31st December 2021. Annual outputs and citations were identified from the WOS citation reports. CiteSpace and VOSviewer were adopted for bibliometric analysis and visualization that included the distribution of countries/regions, organizations, authors and journals, and an analysis of co-cited references and keywords.

**Results:**

A total of 1,638 documents were retrieved for bibliometric analysis, yielding 34.29 citations per document. Publications have increased over the past two decades, reaching 177 outputs in 2019. Our database encompasses 71 countries/regions, 2,347 organizations, and 7,040 authors. The United States ranks first in terms of scientific activity with 604 publications (36.87%). We have identified the University of Sydney as the most prolific organization (53 publications). J. T. Hanlon, J. H. Gurwitz, D. O'Mahony, and G. Onder are the most influential researchers in terms of publications and citations. The Journal of the American Geriatrics Society ranks first with 89 (5.43%) papers. In terms of major research directions, three topics have been identified from co-cited reference and keyword analysis: (1) estimation of the prevalence and variables associated with polypharmacy and potentially inappropriate medication; (2) analysis of interventions involving pharmacists and the associated impact; (3) patient experience and perception associated with medication use or pharmaceutical care.

**Conclusion:**

Research on medication safety for older adults has progressed significantly over the past two decades. The United States, in particular, has made important contributions to this field. Polypharmacy and potentially inappropriate medication use, interventions involving pharmacists, patient experience and perception represent the current focus of research. Our findings suggest that these directions will continue as research hotspots in the future.

## 1. Introduction

Medication safety has been identified as a priority in healthcare systems worldwide, in line with the third World Health Organization (WHO) Global Patient Safety Challenge: Medication Without Harm (1). Medication issues, including errors and error-related adverse drug events (ADEs) are responsible for substantial patient harm (2). Furthermore, medication issues can result in morbidity, hospitalization, increased healthcare costs, and death. Current estimates identify 2.5% of all hospitalizations as medication-related (3), which represents 6.1 emergency department (ED) visits per 1,000 population annually, 38.6% result in hospitalization in the US (4), and more than *e*94 million of the total costs associated with preventable medication-related hospital admissions in the Netherlands (5). The safety of medication is of prime concern to healthcare.

Prevention of damage due to medication mismanagement is a growing challenge in clinical practice, especially for older adults who are subject to chronic care and the prescription of more medications (6, 7). A total of 193 international studies have revealed the prevalence of multi-morbidity (two or more chronic conditions) in a population with mean age ≥74 years (67.0%) and 59–73 years (47.6%) (8). In Europe, ca. 32% of older people take five or more daily medicines (9). Multiple medications in older adults are likely to increase the risk of falls, frailty, cognitive impairment, hospitalization, and ultimate death (10). A longitudinal study in North America showed that one additional medication can increase the risk (by 11%) of frailty for older adults during an 8-year follow-up (11). Moreover, a cohort study has suggested that over 3% of deaths occurred of older patients prescribed with an additional medicine (12).

Given the high risk associated with medication in older people, safe medication is now an important feature of clinical research. The American Geriatrics Society is actively developing the Beers Criteria as a tool to assist caregivers in evaluating the safe, effective, and rational use of drugs for older adults (13–16). The Irish group led by P. Gallagher has devised the Screening Tool of Older Person's Prescriptions (STOPP) and Screening Tool to Alert Doctors to Right Treatment (START) as means of flagging potentially inappropriate medicines (PIMs) for older people (17). These criteria serve to guide interventions and evaluate the therapeutic outcome of prescribing patterns (18, 19). Furthermore, studies directed at safe medication have considered drug-related problems (DRPs) (20, 21), risk factors for ADEs (22), relative adverse health outcomes (12), as well as interventions and effects (18). Work has also examined specific diseases, for example, cancer (21), dementia (20, 23), and heart failure (24).

With the increasing focus of medical care on the safe treatment of older patients, studies of medication safety are burgeoning. It is difficult for researchers to have a full understanding of the current status and development trends. The available literature on medication safety in older adults has focused on polypharmacy management (25, 26), adjudication methods for capturing ADEs (27), strategies for improving medication adherence (28), the prevalence of ADEs (20) and PIMs use (29, 30), and clinical outcomes associated with medication regimen complexity (31, 32). These studies only considered a few aspects of drug safety in older adults using traditional literature review methods. Eshetie et al. (33) have reported four annual high-quality studies that highlight pertinent topics related to the safe use of medications in older adults. However, they did not provide a comprehensive overview of developments in this area. Huang et al. and Giannetta et al. have assessed the literature on medication errors and ADEs using a bibliometric approach (34, 35), but these studies did not focus on an older population. Currently, there has been no attempt to map out the entire field in a systematic manner to summarize important aspects of medication safety research in older adults.

Bibliometrics is the analysis of published academic literature to track developments in a field over a defined period (36, 37). It is an effective methodology to assess the impact of publications and highlight research hotspots, trends, and collaborations (34–37). The objective of this study is to conduct a bibliometric analysis of studies that have specifically considered medication safety for older adults, and illustrate the research landscape in an exploration of significant advancements, hot topics and emerging trends. This paper explores the following five questions: (1) What is the global publication trend related to geriatric medication safety research? (2) Which countries or regions have been dominant in this field? (3) Which organizations, authors, and journals are the most influential? (4) What are currently the most important knowledge bases and research hotspots? (5) What are the future development trends, and what does this mean for academic researchers and healthcare providers?

**Figure 1:**
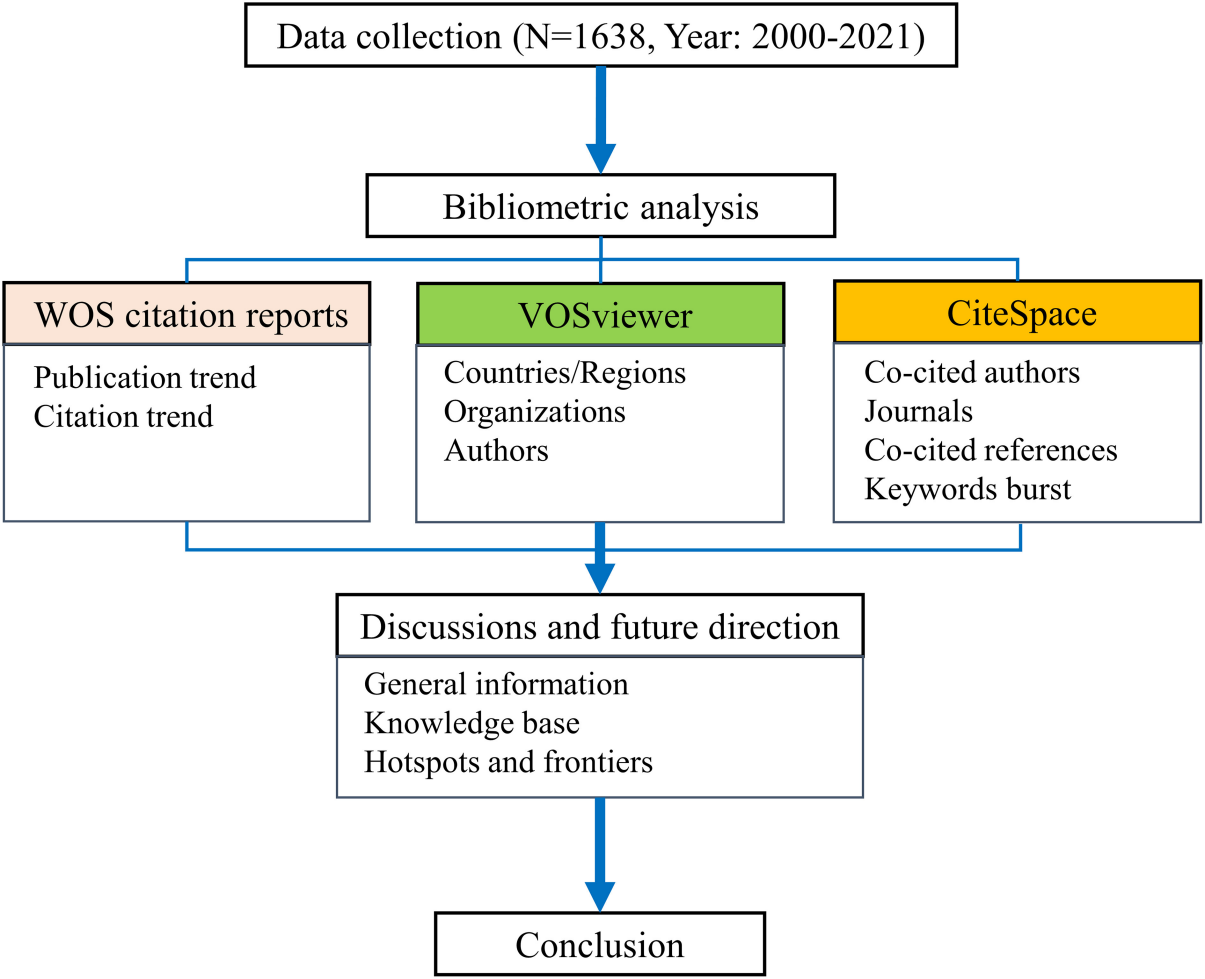
The diagram of article structure.

This article is organized as follows (see Figure 1): in Materials and Methods, we discuss data collection and statistical analysis methods; in Results, we present our analysis of the selected literature, including trends related to publication and citation, distribution of countries/regions, organizations, authors and, journals, reference, and keyword analysis; in Discussion, we provide an overview of the findings of this research, and propose future research directions; in Conclusions, we summarize the state-of-the-art in this field.

## 2. Materials and methods

### 2.1. Data collection and search strategy

This study retrieved literature from the database of the Web of Science Core Collection (WOSCC), which includes the Science Citation Index-Expanded (SCI-E) and the Social Science Citation Index (SSCI). The search strategies were devised following an overview of the literature dealing with medication safety for older adults, optimizing the search using the following search terms: (1) medicine safety topic, including “medication safety,” “medication errors,” and “adverse drug events”; (2) terms for older adults, including “older adults,” “the aged,” “the elderly,” “older people,” “older patient,” and “geriatric.” The search considered articles published from 1st January 2000 to 31st December 2021. We limited the source material to scientific articles and reviews in English. The search strategy adopted in this study is presented in Table 1. In total, 1,638 references were retrieved for bibliometric analysis, of which 1,293 were articles, and 345 were reviews.

**Table 1:** Search strategy for the study of medication safety in older adults.

**Item**	**Content**		
Data sources	WoSCC (SCI-E, SSCI)		
Search strategy	#1	356,485	TS = (“the aged” OR “the elderly” OR “older adults” OR “older people” OR “older patient^*^” OR geriatric)
	#2	20,780	TS = (“medication safety” OR “drug safety” OR “safe medication” OR “medication security” OR “safe drug use” OR “medicine safety” OR “safely use of medicine” OR “drug use errors” OR “medication errors” OR “adverse drug events” OR “medication adverse events” OR “drug related events” OR “medicine related events” OR “medication related events” OR “drug related problems” OR “medicine related problems” OR “medication related problems”)
	#3	39,178,204	DOP = (2000-01-01/2021-12-31)
	#4	48,075,922	DT = (Article OR Review)
	#5	63,403,804	LA = (English)
	#6	1,638	#1 AND #2 AND #3 AND #4 AND #5

### 2.2. Data analysis

Data analysis was conducted using CiteSpace and VOSviewer software. CiteSpace is a bibliometric visualization software that facilitates an analysis of cooperative networks, co-occurrence, co-citation, and the clustering of searching documents, and is also capable of building dual-map overlays of publication journals (38). VOSviewer is free software that enables the analysis of literature data and creating scientific networks (39). In our work, annual trends of publications and citations were identified, as well as the distribution of countries/regions, organizations, authors, and journals. In addition, an analysis of co-cited references and keywords was carried out to reveal the research hotspots of medication safety in older adults. The strategies adopted for bibliometric analysis incorporated: (1) the annual trends of publications and citations generated from the citation reports of WOS; (2) the distribution of countries/regions, organizations, and authors collected using the network map of VOSviewer; (3) the co-cited author networks, co-cited journal networks, dual-map overlays of journals, co-cited reference networks, and keyword bursts analyzed using CiteSpace.

## 3. Results

### 3.1. Temporal trends of publications and citations

**Figure 2:**
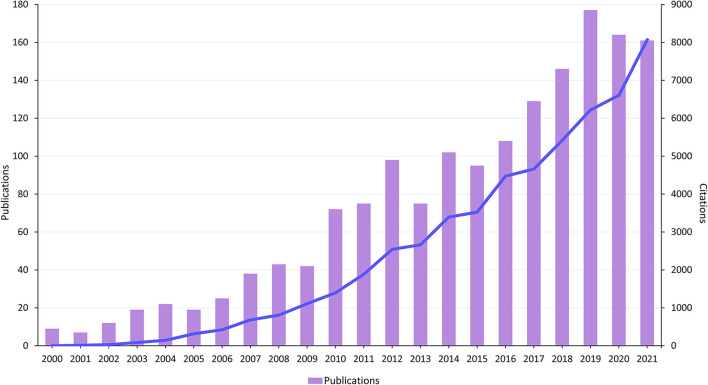
The number of publications and citations from 2000 to 2021.

Over the period 2000 to 2021, the number of records in the WOSCC that defined strategies for safe medication of older adults was 1,638, contributing to 56,172 citations and an average of 34.29 per publication. As shown in Figure 2, the number of studies published increased from 9 in 2000 to 161 in 2021, indicating a surge of activity in this field. The year 2009 represents something of a watershed in terms of publications with a 5-fold increase in papers pre- (23.6 normalized per annum) and post- (116.8) 2009 with the highest number (177) appearing in 2019. The associated number of citations has grown steadily, consistent with the number of publications. In 2021, the number of citations reached 8,056.

### 3.2. Distribution of countries/regions and organizations

**Figure 3:**
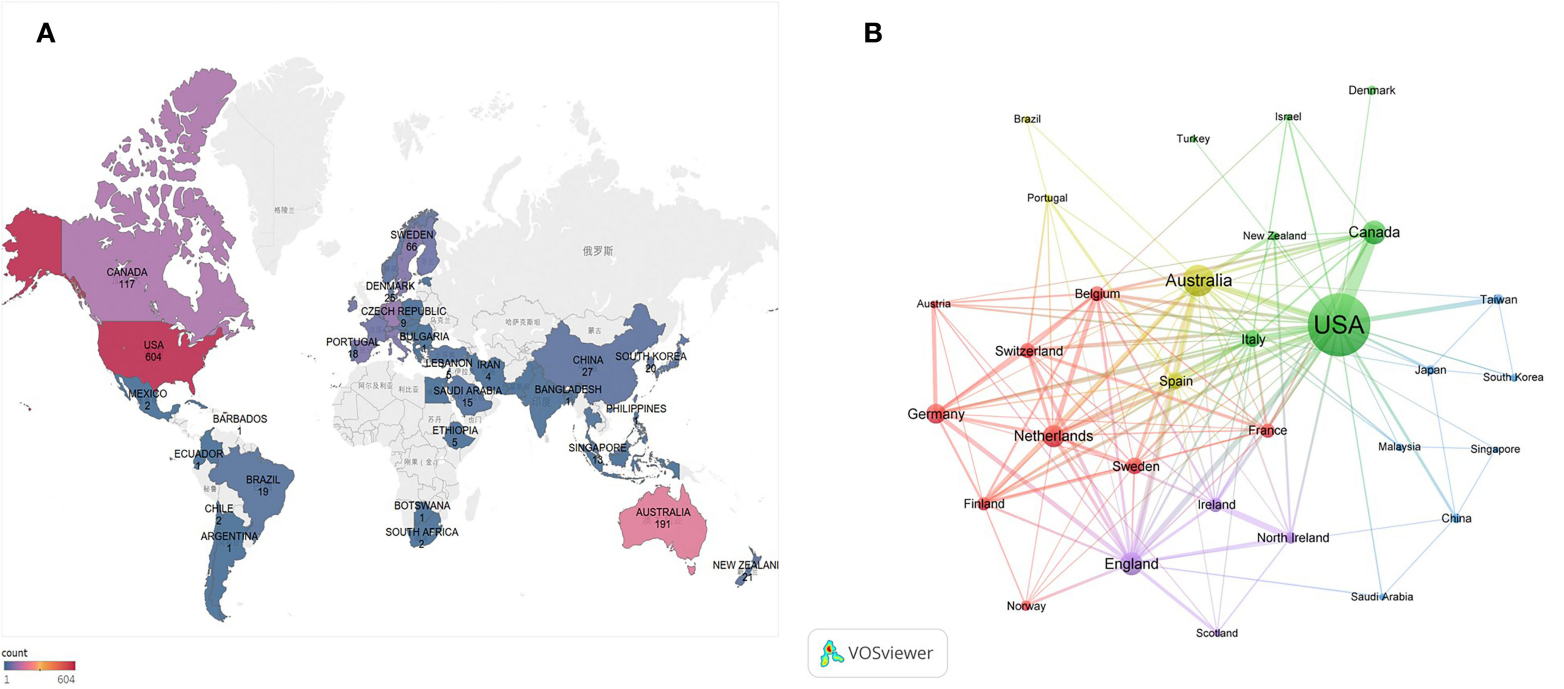
**(A)** World map displays the distribution of medication safety studies in older adults from 2000 to 2021. **(B)** Cooperation network map of countries/regions from 2000 to 2021.

The global distribution of published articles is shown in Figure 3A. A total of 71 countries/regions participated in medication safety research related to older adults. The top 10 countries/regions are listed according to the number of published papers in Table 2. The United States published most articles (*n* = 604), followed by Australia (*n* = 191), and Canada (*n* = 117). Asian countries do not appear in the top 10 of publishing countries. Regarding country/region cooperation, Figure 3B demonstrates the close cooperative relationship between several countries/regions. The link strength in the network map indicates the intensity of the cooperation between nodes. The United States, England, and Australia have the highest total link strength (168, 120, and 113 times, respectively), indicating the level of international cooperation.

**Table 2:** Top 10 countries/regions and organizations in terms of publications from 2000 to 2021.

**Rank**	**Country/region**	**Count (%)**	**Organization**	**Count (%)**
1	USA	604 (36.87)	University of Sydney	53 (3.23)
2	Australia	191 (11.66)	University of Toronto	44 (2.69)
3	Canada	117 (7.14)	Monash University	41 (2.50)
4	England	112 (6.84)	University of Pittsburgh	38 (2.32)
5	Netherlands	102 (6.23)	Karolinska Institute	35 (2.14)
6	Germany	86 (5.25)	University of Massachusetts	34 (2.08)
7	Sweden	66 (4.03)	Queen's University Belfast	32 (1.95)
8	Italy	64 (3.91)	Brigham Women's Hospital	30 (1.83)
9	Spain	64 (3.91)	University of Queensland	30 (1.83)
10	Switzerland	55 (3.36)	Duke University	28 (1.71)

**Figure 4:**
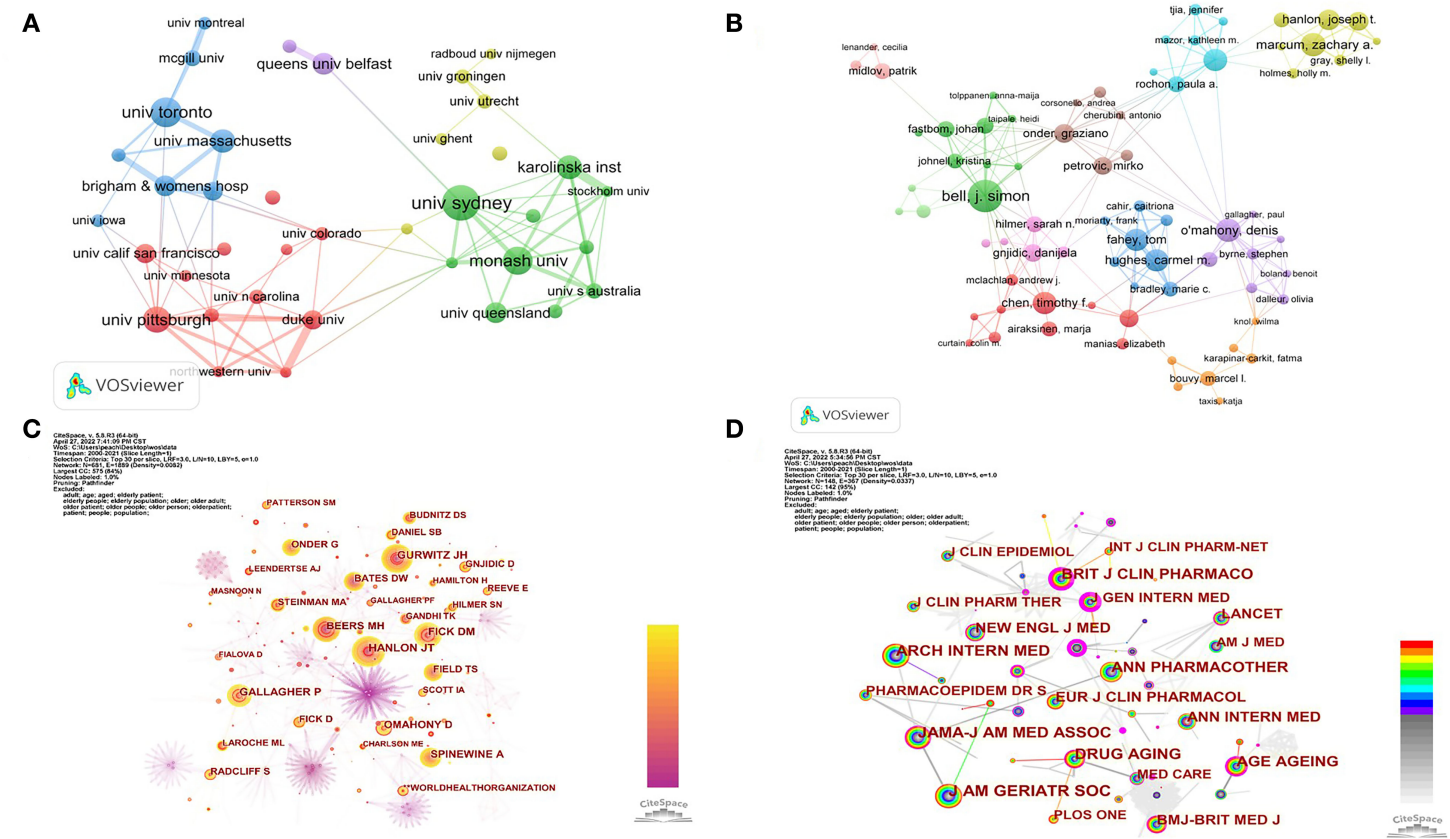
**(A)** Cooperation network map of organizations from 2000 to 2021. **(B)** Cooperation network map of authors from 2000 to 2021. **(C)** Network map of co-cited authors from 2000 to 2021. **(D)** Network map of co-cited journals from 2000 to 2021.

A total of 2,347 organizations contributed to the data presented in the 1,638 research papers. The University of Sydney delivered the greatest number of publications (*n* = 53), accounting for 3.23% of the published literature in this field, followed by the University of Toronto (*n* = 44) and Monash University (*n* = 41); see Table 2. Among the top 10 organizations, four (University of Pittsburgh, University of Massachusetts, Brigham Women's Hospital, and Duke University) are in the United States, three (University of Sydney, Monash University, and University of Queensland) in Australia, and the others (University of Toronto, Karolinska Institute, and Queen's University Belfast) are in Canada, Sweden, and England, respectively. As illustrated in Figure 4A, close cooperation was observed among these scientific research organizations. The three organizations with the highest total link strength were Brigham Women's Hospital (45 times), Monash University (44 times), and Duke University (43 times).

### 3.3. Distribution of authors and co-cited authors

In this study, 7 040 authors contributed to the 1,638 articles on medication safety among older adults, averaging 4.3 authors per article. The 10 most productive authors are identified in Table 3. J. S. Bell from Australia contributed the most studies (*n* = 22), followed by D. O'Mahony (Ireland, *n* = 16) and Z. A. Marcum (United States, *n* = 16). The cooperation network among authors is shown in Figure 4B; the three authors with the highest total link strength are T. Fahey (44 times), D. O'Mahony (38 times), and C. M. Hughes (37 times).

**Table 3:** Top 10 authors in terms of publications and co-cited authors in terms of citations from 2000 to 2021.

**Rank**	**Authors**	**Country**	**Count (%)**	**Co-cited authors**	**Country**	**Citations**
1	J. S. Bell	Australia	22 (1.34)	J. T. Hanlon	USA	364
2	D. O'Mahony	Ireland	16 (0.98)	D. M. Fick	USA	298
3	Z. A. Marcum	USA	16 (0.98)	J. H. Gurwitz	USA	286
4	T. Fahey	Ireland	15 (0.92)	P. Gallagher	Ireland	253
5	C. M. Hughes	Ireland	15 (0.92)	M. H. Beers	USA	234
6	J. H. Gurwitz	USA	15 (0.92)	A. Spinewine	Belgium	215
7	T. F. Chen	Australia	15 (0.92)	D. O'Mahony	Ireland	201
8	J. T. Hanlon	USA	14 (0.86)	D. W. Bates	USA	160
9	G. Onder	Italy	13 (0.79)	S. Radcliff	USA	150
10	K. E. Schmader	USA	13 (0.79)	G. Onder	Italy	146

In order to identify highly cited researchers widely recognized by the academic community, we generated the network map of co-cited authors using CiteSpace. There were 681 nodes and 2,458 links on the network map (Figure 4C). Researchers with more citations tend to have larger nodes. By sorting the data from CiteSpace, we can determine the frequency of co-citation among authors. As shown in Table 3 and Figure 4C, J. T. Hanlon, D. M. Fick, and J. H. Gurwitz have the most citations (364, 298, and 286 citations, respectively) and occupied important node positions in the co-cited network. The top-cited scholars, J. T. Hanlon, J. H. Gurwitz, D. O'Mahony, and G. Onder, also ranked among the top 10 productive authors. In addition, over half of the most cited scholars came from the United States (Table 3).

### 3.4. Distribution of journals and co-cited journals

Over the last two decades, 413 academic journals have published papers in this research field. The top 10 journals published 488 articles, representing 29.8% of the 1,638 studies retrieved (Table 4). *Journal of the American Geriatrics Society* published the most papers (*n* = 89), followed by *Drugs Aging* (*n* = 72), and the *International Journal of Clinical Pharmacy* (*n* = 70). Based on the JCR 2021 standards, only one (*Journal of the American Geriatrics Society*) of the top 10 journals in Table 4 is classified as Q1.

**Table 4:** Top 10 journals in terms of publications and co-cited journals in terms of citations from 2000 to 2021.

**Rank**	**Journal**	**Count (%)**	**JCR (2021)**	**Co-cited journal**	**Citation count**	**JCR (2021)**
1	Journal of the American Geriatrics Society	89 (5.43)	Q1	Journal of the American Geriatrics Society	1,477	Q1
2	Drugs Aging	72 (4.40)	Q2	Archives of Internal Medicine	979	Q1
3	International Journal of Clinical Pharmacy	70 (4.27)	Q4	Journal of the American Medical Association	923	Q1
4	Annals of Pharmacotherapy	41 (2.50)	Q3	Drugs Aging	822	Q2
5	BMC Geriatrics	41 (2.50)	Q2	Annals of Pharmacotherapy	746	Q3
6	European Journal of Clinical Pharmacology	41 (2.50)	Q3	British Journal of Clinical Pharmacology	705	Q2
7	British Journal of Clinical Pharmacology	38 (2.32)	Q2	Age and Aging	700	Q1
8	BMJ Open	35 (2.14)	Q2	New England Journal of Medicine	611	Q1
9	Research in Social Administrative Pharmacy	32 (1.95)	Q2	European Journal of Clinical Pharmacology	592	Q3
10	PLoS ONE	29 (1.77)	Q2	Annals of Internal Medicine	552	Q1

The co-citation relationship among journals is shown in Figure 4D. There were 148 nodes and 656 links in the co-cited network map. *Journal of the American Geriatrics Society*, with the most publications, has been cited most frequently (1,477 times), followed by *Archives of Internal Medicine* (979 times) and *Journal of the American Medical Association* (923 times); see Table 4. The top 10 co-cited journals were cited over 500 times, and more than half of them are classified as JCR Q1.

**Figure 5:**
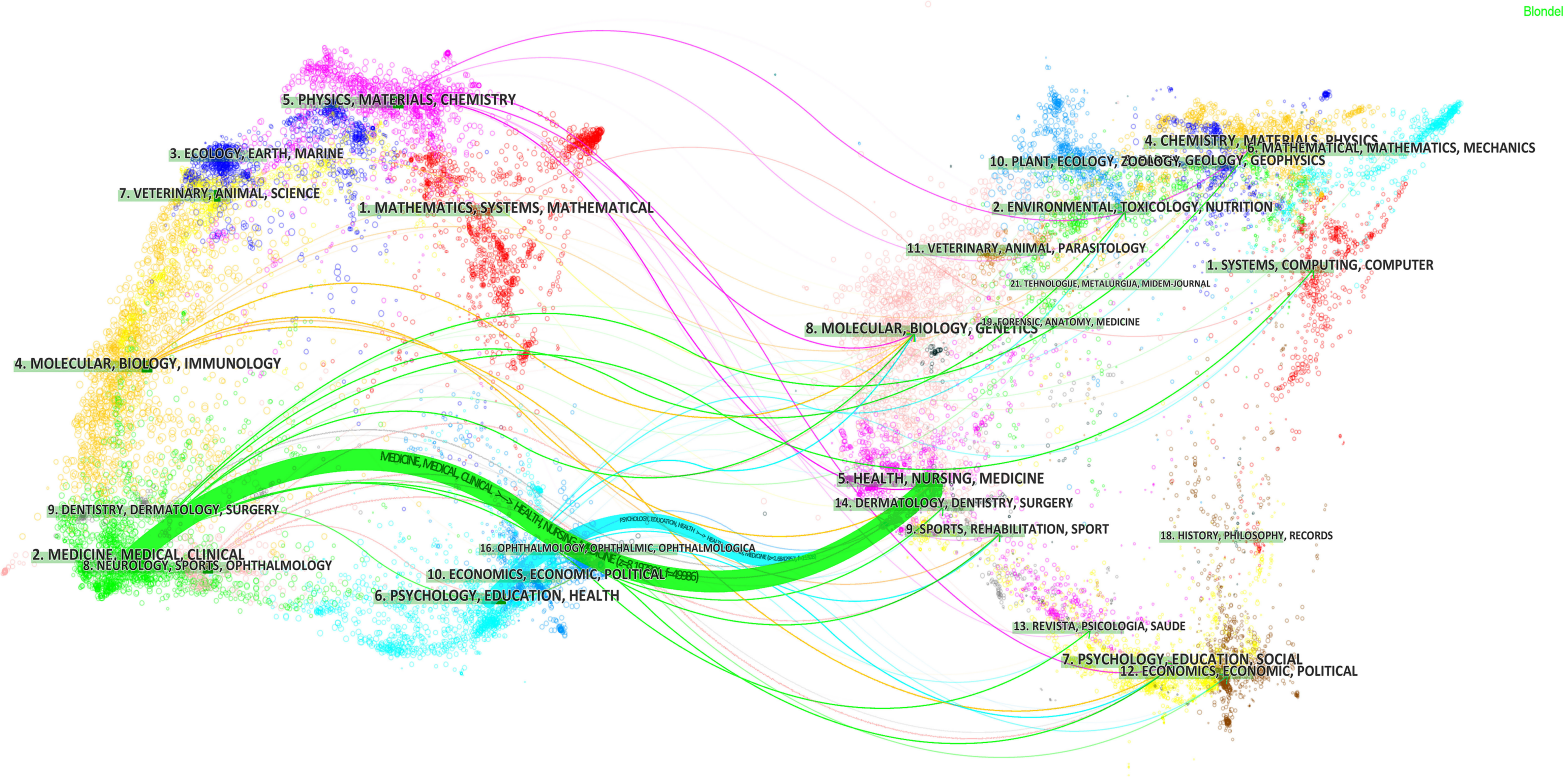
The dual-map overlay of journals publishing studies on medication safety in older adults from 2000 to 2021 [**(Left)**: citing journals; **(Right)**: cited journals].

Figure 5 shows the relationships between the citing journals and cited journals that have published studies of medication safety in older people. Collectively, two primary citation paths (denoted as blue and green in Figure 5) can be discerned from the map. These two paths indicate that most journal articles focus on fields of *medicine, medical, clinical*, and *psychology, education, health*. The majority of articles were cited in journals related to *health, nursing*, and *medicine*.

### 3.5. Analysis of co-cited references

A total of 41,550 valid references cited in the 1,638 records were analyzed to identify underlying knowledge bases and research fronts. The top 12 articles that were cited most frequently are given in Table 5. These highly cited papers were published between 2003 and 2019, and eight were published after 2010. The most highly cited paper was authored by Radcliff et al. (16), published in 2015, with 133 citations. The article by O'Mahony et al. (19), published in 2015, is ranked second with 129 citations. The third most cited (114 citations) describes work by Fick et al. (13), published in 2012. Half of these top-cited papers provide criteria for screening potential inappropriate medication use in older adults (13–17, 19).

**Table 5:** Top 12 cited references in studies on medication safety for older adults.

**Rank**	**Title**	**Year**	**First authors**	**Journal**	**Citation**
1	American geriatrics society 2015 updated beers criteria for potentially inappropriate medication use in older adults	2015	S. Radcliff	Journal of the American Geriatrics Society	133
2	STOPP/START criteria for potentially inappropriate prescribing in older people: version 2	2015	D. O'Mahony	Age and Aging	129
3	American geriatrics society updated beers criteria for potentially inappropriate medication use in older adults	2012	D. M. Fick	Journal of the American Geriatrics Society	114
4	Reducing inappropriate polypharmacy: the process of deprescribing	2015	I. A. Scott	Journal of the American Medical Association Internal Medicine	66
5	American geriatrics society 2019 updated AGS beers criteria for potentially inappropriate medication use in older adults	2019	D. M. Fick	Journal of the American Geriatrics Society	53
6	What is polypharmacy? A systematic review of definitions	2017	N. Masnoon	BMC Geriatrics	52
7	Updating the beers criteria for potentially inappropriate medication use in older adults	2003	D. M. Fick	Archives of Internal Medicine	50
8	Medication use leading to emergency department visits for adverse drug events in older adults	2007	D. S. Budnitz	Annals of Internal Medicine	48
9	Potentially inappropriate medications defined by STOPP criteria and the risk of adverse drug events in older hospitalized patients	2011	H. Hamilton	Archives of Internal Medicine	47
10	Emergency hospitalizations for adverse drug events in older Americans	2011	D. S. Budnitz	New England Journal of Medicine	46
11	Appropriate prescribing in elderly people: how well can it be measured and optimized?	2007	A. Spinewine	Lancet	46
12	STOPP (Screening Tool of Older Persons' Prescriptions) and START (Screening Tool to Alert Doctors to Right Treatment): consensus validation	2008	P. Gallagher	International Journal of Clinical Pharmacology and Therapeutics	46

**Figure 6:**
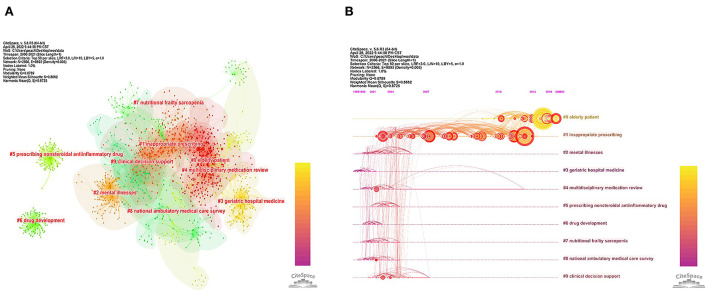
**(A)** Network map of co-cited references from 2000 to 2021. **(B)** Timeline view of the reference co-citation clusters from 2000 to 2021.

The reference co-citation network is shown in Figure 6A. There were 2,566 nodes and 9,892 links on the map. All of the references were divided into 10 clusters labeled with the title terms from the citing articles. The largest cluster was cluster 0 (elderly patient) containing 331 cited papers, followed by cluster 1 (inappropriate prescribing) with 247 papers, and cluster 2 (mental illnesses) with 121 papers. Figure 6B shows the timeline view of the reference co-citation clusters, revealing the temporal characteristics of research hot spots in this field. The development of cluster 3 (geriatric hospital medicine) occurred earliest, suggesting an initial focus on older inpatients. The nodes in cluster 0 (elderly patient) and cluster 1 (inappropriate prescribing) were the largest and most intense, indicating that articles in these clusters were cited the most and received significant attention. Considering the occurrence, node color, and node size of each cluster, we can conclude that cluster 0 (elderly patient) and cluster 1 (inappropriate prescribing) have been the major research fields in medication safety for older adults (Figure 6B).

### 3.6. Analysis of keywords

**Figure 7:**
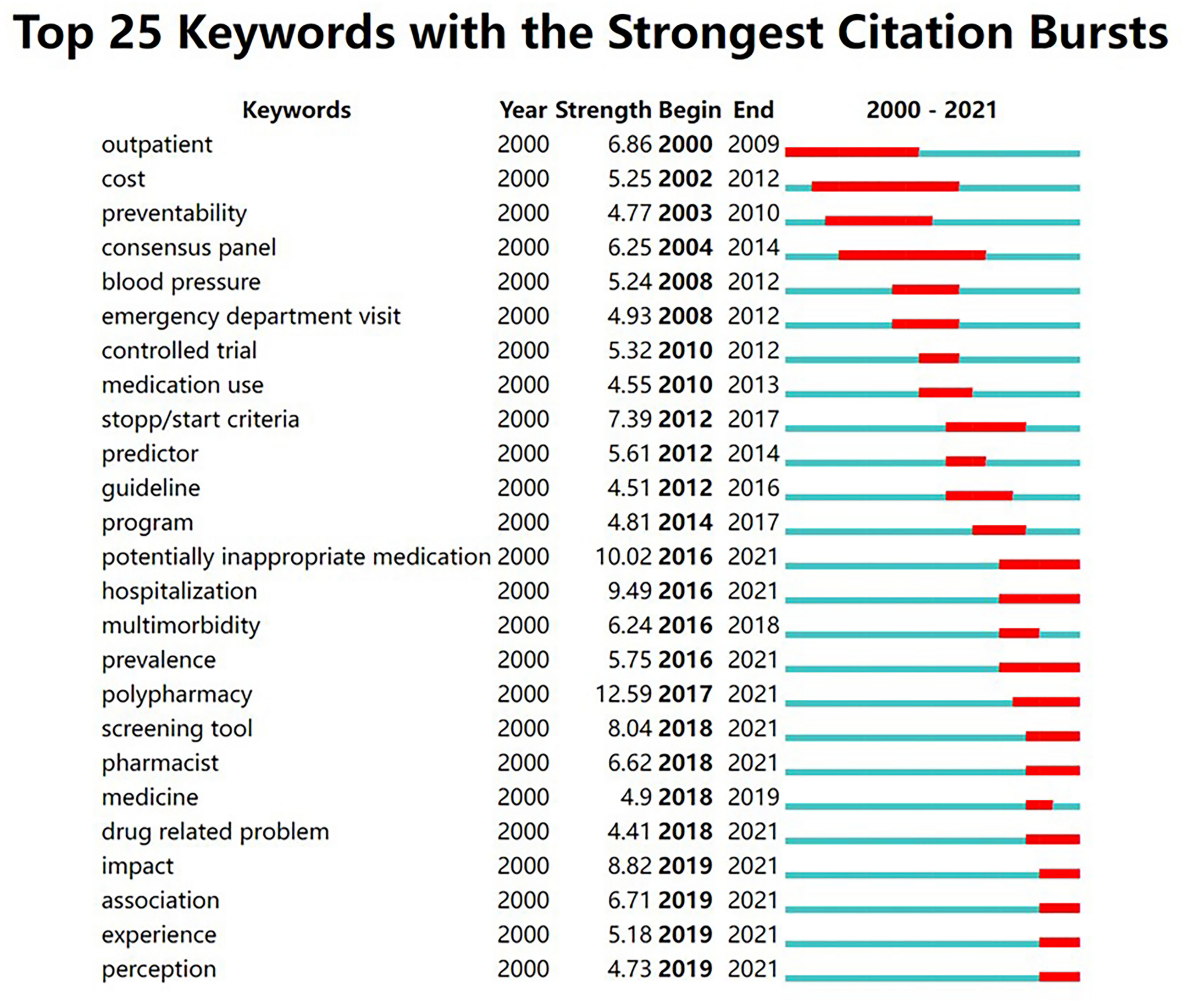
Top 25 keywords with the most robust bursts of research on medication safety in older adults from 2000 to 2021.

Keywords burst represents a rapid increase in occurrence over a period of time, which can serve to pinpoint a research hotspot and frontier in a field (40). We use CiteSpace to identify the burst keywords and their strength and duration over the past decades. Keywords including “older adult,” “older patient,” “older people,” “older person,” “elderly patient,” “elderly people,” “elderly population,” “older,” “age,” “aged,” “adults,” “patient,” “people,” and “population” were omitted from our analysis. The top 25 keywords with the most potent bursts are shown in Figure 7. Among these, “polypharmacy” (12.59), “potentially inappropriate medication” (10.02), and “hospitalization” (9.49) are the top three with the greatest burst strength. As shown in Figure 7, the line on the right represents the duration of occurrence of keywords, and the red coloring indicates the keyword's active period. As can be seen, “potentially inappropriate medication,” “hospitalization,” “prevalence,” “polypharmacy,” “screening tool,” “pharmacist,” “drug related problem,” “impact,” “association,” “experience,” and “perception” are emerging and consistently active keywords between 2016 and 2021. These keywords indicate current research hotspots that also represent future research directions.

## 4. Discussion

### 4.1. General information

This study used bibliometric analysis to illustrate the research landscape and identify hot topics and emerging trends related to the safety of medication for older adults. From 2000 to 2021, the number of papers published in this area has significantly increased. That said, publication numbers declined slightly after 2019. This suggests that medication safety for older adults as a research topic has attracted more attention from scholars in the past two decades, but this activity has slowed somewhat recently. Among the 71 countries/regions participating in this research, the United States has produced the highest number of published articles, three times as many as second-placed Australia (Table 2). This may suggest that the United States attaches particular importance to the safety of medications for the elderly. No country in Asia appears in tabulated data, demonstrating an imbalance in the development of research worldwide. This may be due to the fact that developed countries such as the United States are further advanced with respect to medical concepts and technologies, and have targeted substantial financial support, putting them in a leading position in terms of research output. From a consideration of the cooperation network map (Figure 3B), western countries headed by the United States, England, and Australia have close cooperative relations, while Asian countries such as China, Japan, South Korea, and Saudi Arabia lack international cooperation, especially with western countries, which may compound the uneven development of global research in this field. International collaborations can strengthen research influence and infrastructure by overcoming the lack of suitably trained researchers, low levels of political commitment, and insufficient financial support (41). There is a definite need to strengthen international collaborative research arrangements to facilitate sharing of technical knowledge and promoting in-depth research on drug safety in older adults, especially in developing countries.

Publication numbers of scientific organizations may represent their research capacity. Among the top 10 most productive organizations, four are from the United States, three from Australia, which further confirms the dominant role played by these countries. Our study shows that the most productive organizations are tertiary institutions (Table 2), indicating that universities are at the forefront of research in this area. With the largest number of publications, the University of Sydney has established a pre-eminence in the field of elderly drug safety. In terms of inter-organizational collaboration, cooperation networks are in place between scientific research organizations, notably those from the United States and Australia (Brigham Women's Hospital, Monash University, and Duke University) (Figure 4A). This cross-organizational collaboration further enhances the academic influence of these countries and organizations.

Authors from different countries/regions and organizations have made noteworthy contributions to research in this field. We have identified influential authors through network and co-citation analysis. Our analysis has indicated that the three authors (T. Fahey, D. O'Mahony, and C. M. Hughes) with the strongest collaborative networks are all from Ireland (Figure 4B, Table 3). A greater degree of collaboration was observed for researchers from the same countries/regions when compared with those based in different countries/regions. Regional-specific collaboration facilitates scholar interchange opportunities and inter-institutional support. Our findings further demonstrate the need to strengthen cooperation and exchanges and the importance of cross-regional cooperation. Combined with the number of publications and co-citations, we can identify four scholars (J. T. Hanlon, J. H. Gurwitz, D. O'Mahony, and G. Onder) as influential researchers in the field of medication safety for the elderly. They are all ranked in the top 10 authors in terms of publications and citations (Table 3). The work of J. T. Hanlon addresses medication management in clinically complex older adults, ADEs, and inappropriate drug prescription for the elderly (42–44). J. H. Gurwitz has examined risk factors and strategies for detecting ADEs among older groups (45, 46). D. O'Mahony has established criteria to reduce inappropriate prescriptions for older people (17, 19, 47). The research of G. Onder is directed at effective strategies for optimizing geriatric pharmacotherapy (48, 49). These scholars are considered world leaders in this realm of research, and their studies will continue to ensure safe medication for older adults.

Regarding journals, *the Journal of the American Geriatrics Society* is the most influential both in terms of number of publications and citations, especially citation frequency which is much higher than other journals (1,477 times), as shown in Table 4, indicating that it has the highest recognition and authority in the research on drug safety for older adults. Our study shows that none of the top 10 productive journals with the exception of the *Journal of the American Geriatrics Society* are classified as JCRQ1. In terms of co-citations, more than half of the top 10 journals are JCRQ1 journals (Table 4). Publication in high profile journals ensures that the work has a wide reach in terms of influence. Our findings point to a need for more studies in JCRQ1 journals in order to promote research activity in geriatric drug safety. The information flow to and between journals was analyzed using a dual-map overlay, which revealed trends of the scientific portfolio in the overall visualization. The published studies mainly targeted journals in the fields of *medicine, medical, clinical*, and *psychology, education, health*. These journals, in turn, mostly cited journals from *health, nursing, and medicine*. It can be assumed that future developments in this area are likely to appear in the listed journals. These results can serve as a guide for potential authors in choosing where to submit manuscripts.

### 4.2. Knowledge base

The papers frequently co-cited by scholars can be considered the knowledge base for a specific research field (50, 51). These co-cited studies are the cornerstones, laying the foundation for developing new research. Thus, we analyzed the co-citation references to evaluate the knowledge base on medication safety in older adults.

In our study, six of the 12 top-cited papers provided screening tools for PIMs used for older adults (Table 5). The tools included the American Geriatrics Society Beers Criteria (ACS Beers Criteria) (13–16), the STOPP and START (17, 19). The ACS Beers Criteria lists PIMs or medication classes that should be avoided in older adults (15). The STOPP specifies clinically significant criteria for assessing potentially inappropriate prescriptions, and the START provides evidence-based prescription indicators for commonly encountered diseases in older patients (19). An article on the 2015 version of Beers Criteria by Radcliff et al. (16) and the updated STOPP and START criteria reported by O'Mahony et al. (19) have received the most citations in our bibliometric study, which identifies those papers which have received significant international attention. Two of the highly cited papers (52, 53) published by Budnitz et al. have considered emergency department (ED) visits for ADEs in older adults. One study reported the number and risk of ED visits related to ADEs (52), and the second study established the frequency and rates of hospitalization after ED visits for ADEs (53). Scott et al. (54) proposed a five-step de-prescribing protocol for reducing inappropriate polypharmacy in older adults. Masnoon et al. (25) published a systematic review in 2017 (with 52 co-citations) that summarized the definition of “polypharmacy,” and established comorbidity-related medication as “appropriate polypharmacy.” The work of Hamilton et al. (55), the ninth most commonly co-cited paper, reported that PIMs defined by new STOPP criteria were associated with avoidable ADEs and contribute to urgent hospitalization in older people with acute illness. Spinewine et al. (56) have examined the definition and categories of appropriate prescriptions in older people, reviewing measures of the appropriateness with an assessment of optimization strategies.

Taking an overview of the literature, these top-cited articles have mainly involved screening tools for PIM use in older people, approaches to reducing inappropriate polypharmacy or prescribing, and risk assessment of ED visits or hospitalization for ADEs. This is essential groundwork that provides guidance and establishes the medication safety research status quo for scholars interested in the subject, as well as establishing the theoretical basis for clinical evidence-based practice.

### 4.3. Hotspots and frontiers

The reference co-citation network maps the research frontier and its knowledge base by constructing the relationship between the citing and co-cited articles (38). Our study has labeled clusters in the reference co-citation network with title terms from citing articles. Compared with the co-cited papers as the knowledge base, these citing articles and terms can be regarded as emerging research subjects in this field. From the timeline view of the reference co-citation clusters (Figure 6B), we have found that articles in cluster 0 (elderly patient) and cluster 1 (inappropriate prescribing) were cited the most. This serves to indicate that inappropriate prescription for older patients is a major concern in this research area. Apart from reference co-citation networks, keyword analysis is crucial for predicting research trends and identifying hot spots. Strong burst keywords are those that have received appreciable attention from the scientific community in a particular period and might therefore represent research frontiers and hotspots (38, 40). Our study shows that the keyword “potentially inappropriate medication” is among the top three with the most robust burst strength, which further confirms that inappropriate prescription and inappropriate medication are the focus of global geriatric drug safety concerns. Based on the keyword bursts period, we have found keywords like “polypharmacy,” “pharmacist,” “impact,” “experience,” and “perception” which have exhibited recent bursts (Figure 7). According to these keyword bursts, the following development trends in medication safety research in older adults apply:

(1) Estimating the prevalence of, and variables associated with, polypharmacy and potentially inappropriate medication use.

Older patients with several co-morbidities frequently take multiple medicines, commonly termed polypharmacy, which is defined as taking five or more medications daily (25). Medications, where the risks associated with their use outweigh the benefits, are considered potentially inappropriate medications (PIMs) (57). Polypharmacy and PIM use are recognized as widespread global issues among the elderly (10, 58). Identifying these issues is the first step to reducing pharmacotherapy-related hazards in this vulnerable population. The prevalence of, or variables associated with, polypharmacy and PIM use in older people have been studied in various countries. Data has shown that polypharmacy is highly prevalent among older adults in 17 European countries (26.3%) and Israel (39.9%) (9). In Ethiopia, polypharmacy and PIM use in older people was recorded at 33 and 37%, respectively (58). Identifying variables associated with polypharmacy and PIM use is essential to identify and monitor those most at risk (9, 58). Polypharmacy predictors involve age, gender, physical inactivity, limitations with daily activities, quality of life and wellbeing, depression, chronic disease, difficulties in taking medication, years of education, and financial circumstances (9). In addition, the consequences of polypharmacy and PIM use in older people have considered adverse health, social, and medication management outcomes (31), such as higher rates of drug related problems (DRPs), hospitalizations, and mortality rates (29, 32). As elderly patients exhibit different clinical characteristics and different medication profiles, further research on polypharmacy and PIMs use in those with specific clinical features is still required. Successful intervention by clinicians to optimize drug therapy management requires a detailed consideration of the diversity of factors and outcomes linked to polypharmacy and PIM use in older adults.

(2) Analyzing interventions involving pharmacists and their impact.

Optimizing drug prescriptions and medication reviews are the principal approaches to reducing PIMs use and preventing medication harm among an aged population (59, 60). As experts in pharmacotherapy, clinical pharmacists undoubtedly have a crucial role in this respect. Studies have been conducted on the involvement of pharmacists in pharmaceutical care for elderly patients (60–63) that have considered various settings such as in-hospital geriatric wards, community pharmacies, and nursing homes. Clinical pharmacists are involved in medication reconciliation (64), identifying DRPs (65), counseling or medication reviews (60), and collaborating with other health care professionals as multidisciplinary team members (61). In a multidisciplinary team, pharmacists offer an additional perspective in the application of medication review and assist other healthcare providers in rational therapeutic decision-making (61, 63). The impact that pharmacists exert on older patients includes optimizing polypharmacy (66), increasing medication appropriateness (61), improving patients' medication adherence (61, 67), reducing occurrence of DRPs (65), decreasing mortality and adverse drug reactions (66, 67), as well as the number of hospitals visits and healthcare costs (26, 60). The literature findings suggest that pharmacists are uniquely qualified to improve quality of medication use and health outcomes. Although their positive role is undeniable, there are still many obstacles for pharmacists to fully contribute to clinical practice, including inadequate support from authorities, other health professionals, and the public, as well as professional encroachment on the medical domain (68). The role of pharmacist participation and relationship with other healthcare professionals warrant future research.

(3) Identifying patient experience and perception associated with medication use or pharmaceutical care.

Patient perception and experience are essential considerations for clinicians when making clinical decisions (59). Patient experiences with drugs incorporate perceived benefits, side effects, burden, expense, prior de-prescribing and other interruptions in treatment, financial toxicity, and out-of-pocket drug expenses (59). The perspectives of older patients on medication use were identified in several studies, including barriers and facilitators encountered during the transition from hospital to home (69), factors potentially contributing to ADEs in ambulatory settings (70), and attitudes toward de-prescribing (71). In addition to patient experience and perceptions of drugs, preferences concerning clinical interventions were essential for clinicians. Important in this respect are the experiences and views of older patients regarding medication reviews and follow-up telephone calls by clinical pharmacists (72), patient preferences for relative de-prescribing authority (73), and factors associated with decision-making in a Medicare Prescription Drug Plans service (74). Patient perception may differ depending on their medical condition and their relationship with the clinician (73). Identifying the older patient's experiences and preferences may provide further insight into current needs and priorities concerning safe medication use, and allow for a better understanding of the effects of clinical interventions (69, 72). These are essential factors in facilitating effective design and implementation of the intervention and improving clinical practice. Our study has revealed that patient perceptions and experiences are increasingly valued in geriatric medication safety research. In future pharmaceutical care of older patients, the psychological and practical barriers patients face in drug therapy management are essential for clinical practice to formulate patient-centered medication safety strategies.

## 5. Conclusion and implications

Based on bibliometrics and visualization methods, this study provides a new perspective on the field of medication safety in older adults from a systematic analysis of the academic literature. The findings can be summarized as follows.

(1) Geriatric medication safety research has been the subject of increasing overall publications, although the number of papers has declined since 2019.

(2) Global research in this field has exhibited uneven development in terms of geographical distribution. Western countries headed by the United States have made the most contributions to geriatric medication safety research and have close cooperative relations. Asian countries lack international cooperation and need to strengthen their output. In terms of organizational distribution, the University of Sydney is the most influential organization in the field.

(3) The Journal of the American Geriatrics Society is the most widely researched journal on geriatric medication safety. Four scholars (J. T. Hanlon, J. H. Gurwitz, D. O'Mahony, and G. Onder) have been identified as the most influential researchers based on their publications and citations. Their work has contributed significantly to advancements in medication safety for the elderly.

(4) Highly-cited literature analysis have established screening tools for PIM use in older people, approaches to reducing inappropriate polypharmacy or prescribing, and risk assessment of ED visits or hospitalization for ADEs as forming the current knowledge base for geriatric drug safety research. Combined with the strong burst keyword analysis, polypharmacy, inappropriate prescription and inappropriate medication were identified as research hotspots in this field.

(5) Future development trends in medication safety research for older adults should focus on in-depth studies that tackle the research hotspots. Polypharmacy and PIM use remain serious threats to elderly drug safety, and the prevalence and associated variables among elderly patients with specific clinical features should be the subject of future work. In addition, research on drug safety in the elderly must continue to address interventions involving pharmacists to promote the practice of drug safety for older patients. As healthcare increasingly shifts toward patient-centered care, the experience and perception of elderly patients will play a crucial role in addressing drug safety issues. Continued exploration the experience and perception of elderly patients will facilitate future advances in the field.

In accordance with these findings, we can comment on important managerial implications. Firstly, measures are required to encourage cross-regional and cross-organizational collaboration, especially among developing countries and their organizations. This will promote sharing of technical knowledge and enhance current understanding of medication safety in the elderly. Secondly, it is recommended that healthcare providers use the screening tool in the latest guidelines, and carefully consider the diversity of factors and outcomes linked to polypharmacy and PIM use in older adults when deciding optimal drug therapy management interventions. Thirdly, measures should to be taken to reduce barriers for pharmacists to participate in health care inter-professional teams, and to maximize the positive contribution of pharmacists in medication management for older people. Finally, in addition to optimizing the appropriate medication regimen, the experience of older patients and their perception of drugs and pharmaceutical care must not be ignored in medication management.

## 6. Strengths and limitations

This study uses a systematic bibliometric approach to analyze studies on medication safety for older adults over the past two decades. Publication trends, country/regional distribution, active organizations, productive authors, and core journals have been identified in this paper, as well as the knowledge base, research hot topics, and emerging trends. Our findings can provide scientific researchers with a panoramic view of this area and critical references for future directions, while informing decisions in selecting and designing clinical practice interventions. We should note potential limitations. Firstly, the WOS database was adopted for this study, which is recognized as a more accurate and reliable tool than Scopus and other databases (75). That said, as only English language articles and reviews have been considered, some influential articles may have been overlooked. Secondly, the results presented in this paper were generated using CiteSpace and VOSviewer, and these algorithms may be prone to bias. Although the retrieval strategies were based on literature dealing with medication safety for older adults, some relevant articles may have been missed as the topic draws on a broad content, which may have led to some bias in the ultimate visualization.

## Author contributions

CX: conceptualization, methodology, data curation, and writing—review and editing. YG: conceptualization, methodology, supervision, project administration, funding acquisition, and writing—review and editing. YW: methodology and data curation. FN and YL: data curation and review. All authors contributed to the article and approved the submitted version.
